# Reduced Reliability of Procalcitonin (PCT) as a Biomarker of Bacterial Superinfection: Concerns about PCT-Driven Antibiotic Stewardship in Critically Ill COVID-19 Patients—Results from a Retrospective Observational Study in Intensive Care Units

**DOI:** 10.3390/jcm12196171

**Published:** 2023-09-24

**Authors:** Giancarlo Ceccarelli, Francesco Alessandri, Giuseppe Migliara, Valentina Baccolini, Giovanni Giordano, Gioacchino Galardo, Carolina Marzuillo, Corrado De Vito, Alessandro Russo, Massimo Ciccozzi, Paolo Villari, Mario Venditti, Claudio M. Mastroianni, Francesco Pugliese, Gabriella d’Ettorre

**Affiliations:** 1 Hospital Policlinico Umberto I, 00161 Rome, Italy; giancarlo.ceccarelli@uniroma1.it (G.C.); giordano.gj@gmail.com (G.G.); giuseppe.migliara@uniroma1.it (G.M.); valentina.baccolini@uniroma1.it (V.B.); gioacchino.galardo@gmail.com (G.G.); mario.venditti@uniroma1.it (M.V.); claudio.mastroianni@uniroma1.it (C.M.M.); f.pugliese@uniroma1.it (F.P.); gabriella.dettorre@uniroma1.it (G.d.); 2 Department of Public Health and Infectious Diseases, University of Rome Sapienza, 00185 Rome, Italy; carolina.marzuillo@uniroma1.it (C.M.); paolo.villari@uniroma1.it (P.V.); 3 Intensive Care Unit, Department of General, Specialistic Surgery, University of Rome Sapienza, 00185 Rome, Italy; 4 Infectious and Tropical Disease Unit, Department of Medical and Surgical Sciences, ‘Magna Graecia’ University of Catanzaro, 88100 Catanzaro, Italy; a.russo@unicz.it; 5 Unit of Medical Statistics and Molecular Epidemiology, University Campus Bio-Medico of Rome, 00128 Rome, Italy; massimo.ciccozzi@unicampus.it

**Keywords:** procalcitonin, PCT, biomarker, SARS-CoV-2, COVID-19, ICU, intensive care unit, critically ill

## Abstract

Background: The aim of this study was to assess whether procalcitonin levels is a diagnostic tool capable of accurately identifying sepsis and ventilator-associated pneumonia (VAP) even in critically ill COVID-19 patients. Methods: In this retrospective, observational study, all critically ill COVID-19 patients who survived for ≥2 days in a single university hospital and had at least one serum procalcitonin (PCT) value and associated blood culture and/or culture from a lower respiratory tract specimen available were eligible for the study. Results: Over the research period, 184 patients were recruited; 67 VAP/BSI occurred, with an incidence rate of 21.82 episodes of VAP/BSI (95% CI: 17.18–27.73) per 1000 patient-days among patients who were included. At the time of a positive microbiological culture, an average PCT level of 1.25–3.2 ng/mL was found. Moreover, also in subjects without positive cultures, PCT was altered in 21.7% of determinations, with an average value of 1.04–5.5 ng/mL. Both PCT and PCT-72 h were not linked to a diagnosis of VAP/BSI in COVID-19 patients, according to the multivariable GEE models (aOR 1.13, 95% CI 0.51–2.52 for PCT; aOR 1.32, 95% CI 0.66–2.64 for PCT-72 h). Conclusion: Elevated PCT levels might not always indicate bacterial superinfections or coinfections in a severe COVID-19 setting.

## 1. Introduction

The management of superinfections was one of the key issues in Intensive Care Units (ICUs) throughout the pandemic. In particular, bacterial secondary infections have been reported to have a variable incidence depending on the local epidemiology and clinical setting, and to severely affect morbidity and mortality in critically ill COVID-19 patients [1,2,3,4]. The challenge for clinicians was to identify superinfections early, quickly, and correctly due to the possible syndromic overlap between bacterial and SARS-CoV-2-related sepsis. In this sense, monitoring procalcitonin (PCT) levels has been widely adopted in clinical practice even during COVID-19, considering that, in the pre-pandemic era, it represented a reliable laboratory tool in diagnosing and managing bacterial infections, as well as guiding antibiotic therapy [5,6]. In fact, PCT is a peptide precursor of the hormone calcitonin, which is primarily produced by the C-cells of the thyroid gland. Its blood levels selectively increase in response to systemic bacterial infections in COVID-19-negative patients, and, for this reason, is used as a biomarker for differentiating between bacterial and viral infections, as viral infections typically do not lead to significant increases in procalcitonin levels [6]. In fact, its utilization relies on two fundamental assumptions: first, that viruses indirectly inhibit PCT production by inducing inhibitory interferons, primarily interferon-γ (IFN-γ) [7,8,9,10,11], and, second, that bacteria directly stimulate the expression of PCT through lipopolysaccharide and indirectly via the induction of proinflammatory cytokines such as interleukin-1β (IL-1β), IL-6, and tumor necrosis factor α (TNF-α) [12,13,14,15]. Moreover, PCT can also assist clinicians in determining the severity of the infection and monitoring the response to treatment [6]. In the clinical practice, the use of PCT was approved by the Food and Drug Administration (FDA) to guide antibiotic treatment in sepsis and lower respiratory tract infections for SARS-CoV-2-uninfected patients [16]. Moreover, PCT-guided antibiotic stewardship has been shown to be useful in optimizing antibiotic prescription with an improvement in clinical outcomes, a reduction in potential side-effects, and better control over the emergence of antibiotic resistance [5]. The sudden appearance of the SARS-CoV-2 pandemic at the end of 2019 put health services under great pressure, requiring maximum clinical effort in the absence of sufficient scientific evidence for the effective management of patients with COVID-19. Especially in the early pandemic phases, patient management was based on the use of already existing diagnostic, clinical monitoring, and therapeutic approaches brought about by settings different from that of SARS-CoV-2 infection. The validation of such approaches in the COVID-19 setting necessarily took place on the pandemic battlefield, as there was no time for validation in clinical studies.

The purpose of this study was to evaluate whether procalcitonin levels could be a diagnostic tool capable of correctly marking sepsis and ventilator-associated pneumonia (VAP) even in the setting of critically ill COVID-19 patients.

## 2. Materials and Methods

### 2.1. Design of the Study, Population, Settings, Data Collection, and Outcomes

**Figure 1:**
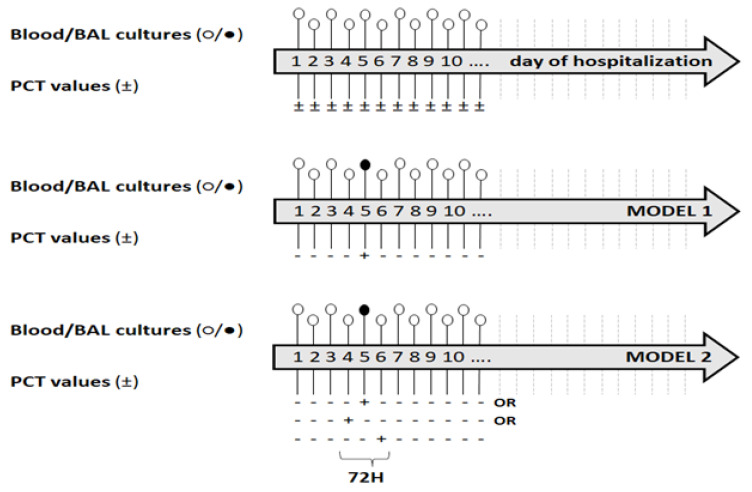
Design of the study and models of analysis. Legend—PCT: (+) positive, (−) negative. Blood cultures: ○ normal value, ● pathological value.

This study is a “real-life” retrospective, observational study conducted on data from the healthcare-associated infection (HAI) surveillance system active in the ICUs of the Umberto I University Hospital of Rome (Italy). The study design was based on matching the available PCT values to blood culture and cultures *of* bronchoalveolar lavage (BAL) collected at the same time in critically ill COVID-19 patients, to evaluate whether PCT values increased due to synchronous clinically significant bacteremia and/or VAP, as expected in no-COVID-19 setting (Figure 1). All critically ill COVID-19 patients who recovered for more than 2 days in the 2 ICUs of the Umberto I University Hospital of Rome (Italy) from March 2020 to February 2021 with at least one serum PCT value and related blood culture/BAL culture available were eligible for the analysis.

The sources for patient data were medical records stored in the electronic information system of the ICU involved. The variables considered included: past clinical history (comorbidities), current clinical history, treatment, ventilation parameters, and laboratory and microbiological data. The study’s primary endpoint was to evaluate positive and negative predictive values (PPV and NPV) of PCT test in identifying an underlying VAP/BSI on the same date of the test. The secondary endpoint was the same evaluations using a model that takes into consideration a 72 h window (i.e., considering as positive the PCT test in the 24 h preceding or following a positive PCT test) (Figure 1).

### 2.2. Diagnosis of SARS-CoV-2 Infection and COVID-19-Related Pneumonia, and COVID-19 Treatments

The diagnosis of SARS-CoV-2 infection was defined as at least one positive oro-nasopharyngeal swab or bronchoalveolar liquid for SARS-CoV-2 E and S gene by a RT-PCR. Stratification of SARS-CoV-2 infection severity was based on World Health Organization (WHO) criteria [17]. COVID-19-related pneumonia was diagnosed by high-resolution CT/non-contrast enhanced chest CT. The patients were treated with ad interim routinely used therapy as suggested by the provisional guidelines of the Italian Society of Infectious and Tropical Diseases (SIMIT) and the Italian Medicine Agency (AIFA) [18]. All patients included in the study were supported by oxygen therapy delivered via invasive mechanical ventilation.

### 2.3. Clinical Evaluation, PCT Dosage, and Microbiological Analysis

Multiparametric monitoring and clinical evaluations of critically ill patients were carried out continuously: subjects who developed clinical signs suggestive of sepsis or persistent fevers, or deteriorated clinically, without other plausible explanations, were also subjected to microbiological examination and PCT dosage, as part of an overall evaluation for the differential diagnosis.

Quantitative analysis of serum PCT was performed routinely at least every 72 h, and every 24 h in case of suspected superinfection, using BRAHMSTM PCT sensitive KRYPTOR^®^ immunoassay (ThermoFisher, Hennigsdorf, Germany). Plasma levels of >0.5 ng/mL were interpreted as abnormal and suggested a possible bacterial superinfection. The half-lifetime of PCT was considered to be 22.3–28.9 h (25–50 percentiles) with no significant difference due to gender, age, and renal dysfunction [6,19].

Three sets of blood cultures (fill volume 10 mL/bottle) and a semiquantitative culture from a lower respiratory tract specimen (such as distal protected aspirate) were drawn at admission from every critical COVID-19 patient transferred to the ICU from the isolation ward. Blood and respiratory tract specimen were also retaken for cultures if the patient developed clinical signs of sepsis, had persistent fever, or deteriorated clinically, without other plausible explanations.

Bacterial identification was performed by a matrix-assisted laser desorption/ionization time-of-flight mass spectrometry (MALDI-TOF MS) system (Bruker Daltonik GmbH, Bremen, Germany).

The guidelines of the European Committee on Antimicrobial Susceptibility Testing (EUCAST) were used for the interpretations of isolate susceptibility. [20]

### 2.4. Definition

VAP was defined according to the criteria proposed by the European Center for Disease Control [21]. Sepsis was defined according to SEPSIS-3 criteria and ARDS was identified according to the 2012 Berlin criteria [22,23]. Single cultures positive for coagulase-negative staphylococci were only considered as environmental contaminations and, therefore, considered as negative in terms of infectious events (BSI).

### 2.5. Statistical Analysis

Descriptive statistics were calculated using median and interquartile range (IQR) for non-normal continuous variables and frequencies and proportions for dichotomous and categorical variables. Normality of continuous variables was checked through the Shapiro–Wilk test. To avoid any potential increase of PCT values that could occur during a confirmed VAP/BSI, the seven days following a VAP/BSI diagnosis were excluded from the analysis. At first, PCT values were considered positive for values greater than 0.5 ng/mL. Then, to take into account the repeated PCT measurements for each patient, a multivariable generalized estimating equation (GEE) regression model with a logit link, a binomial error structure, and exchangeable correlation structure (Model 1) was built to explore the association of the exposure of interest (PCT positivity) to the outcome of interest (positive blood culture) through the estimation of its adjusted odds ratio (aOR) and the associated 95% confidence intervals (CI). Variables were included in the model based on expert opinion. The final model included the following variables: sex (dichotomous); age (continuous); comorbidity (dichotomous); and Simplified Acute Physiology Score (SAPS) II (continuous). To also account for possible delays between the PCT blood peak and the blood culture positivity, a second statistical model (Model 2) was built considering as positive the PCT test in the 24 h preceding and following the identification of the PCT positivity (PCT-72 h) (Figure 1). Sensitivity and specificity of PCT and PCT-72 h test in predicting an underlying VAP/BSI were calculated using the GEE regression method for clustered data [24]. In addition, we also estimated adjusted sensitivities and specificities for PCT and PCT-72 h, accounting for the imperfect accuracy of hemoculture to detect micro-organisms in the blood during sepsis as the gold standard [24,25,26]. Given that this adjustment method is unable to account for repeated measures, the adjustment was performed on the standard sensitivity and specificity of PCT, calculated without any further adjustment as a way to study the effect of the imperfect accuracy of the gold standard.

Positive and negative predicted values were calculated using Bayes Theorem, considering the period prevalence of VAP/BSI that registered in our study sample (i.e., 36.41%).

Lastly, as a sensibility analysis, PPVs and NPVs were estimated for increasing PCT and PCT-72 h cut-off values, using an interval of 0.1 ng/mL from 0.1 to 15.0 ng/mL.

All statistical analyses were performed using Stata (StataCorp LLC, 4905 Lakeway Drive, College Station, TX, USA) version 17.0. A two-sided *p*-value < 0.05 was considered statistically significant.

### 2.6. Ethics Committee Approval

The Ethics Committee of Policlinico Umberto I approved the study with number 109/2020. This study follows the relevant EQUATOR network reporting guidelines and STROBE reporting guidelines for observational studies.

## 3. Results

Demographic and clinical characteristics of the patients enrolled were reported in Table 1.

In the study period, 67 instances of VAP/BSI occurred, and an incidence rate of 21.82 episodes of VAP/BSI (95% CI: 17.18–27.73) per 1000 patient-days was observed among patients enrolled. The most frequently isolated strain was *Acinetobacter baumannii* (55.2%), followed by *Klebsiella pneumoniae* (20.9%), Enterococci (13.4%), *Staphylococcus aureus* (9.0%), Enterobacteriaceae other than *K.pneumoniae* (7.5%), and other micro-organisms (4.5%).

Overall, 2044 determinations of PCT were collected over 3070 cumulative days of hospitalization (66.6%): pathological values were reported in 21.7% of cases. In particular, a median of 9 PCT determinations (IQR 5–15) and a median time of 13 days of follow-up (IQR 8–22) was available for each patient enrolled. The average PCT level reported at the moment of a positive culture was 1.25 ± 3.4 ng/mL. On the other hand, PCT was found to be altered in 21.7% of the determinations in subjects without positive cultures, with an average value of 1.04 ± 5.5 ng/mL. Finally, 19 of the 67 VAP/BSI (28.4%) occurred without a PCT evaluation, 15 of which were not even in the previous and following 24 h.

### 3.1. Predictors of VAP/BSI

The multivariable GEE models showed that neither PCT nor PCT-72 h were associated with a diagnosis of VAP/BSI in COVID-19 patients (aOR 1.13, 95% CI 0.51–2.52 for PCT; aOR 1.32, 95% CI 0.66–2.64 for PCT-72 h) (Table 2). No other factor showed any association with the outcome.

### 3.2. Diagnostic Accuracy of PCT and PCT-72 h

The estimation of the accuracy of PCT in discriminating positive cultures had a sensibility of 19.2% (95% CI: 10.1–33.3%) and a specificity of 81.6% (95% CI: 76.4–85.9%). Using the less stringent PCT-72 h as a marker for positive cultures, the sensibility increased to 30.8% (95% CI: 19.7–44.7%), while the specificity remained high (77.0%; 95% CI: 71.3–82.0%). Accordingly, PPVs and NPVs increased from 37.4% (95% CI: 25.9–51.8%) to 43.5% (95% CI: 33.3–54.2%) and from 63.8% (60.5–67.0%) to 66.0% (95% CI: 61.7–70.2%), respectively.

Given the high sensibility of hemocultures in triplicates (97%) and their virtually perfect specificity [20], the adjusted sensitivity and specificity remained virtually identical to the unadjusted ones for both PCT (18.7% and 79.0%, respectively; PPV: 33.9%; PNV: 62.9%) and PCT-72 h (35.7% and 69.3%, respectively; PPV: 30.7%; PNV: 70.2%).

### 3.3. Sensitivity Analysis

Supplementary Table S1 shows the diagnostic accuracy of PCT and PCT-72 h values in predicting an underlying VAP/BSI, considering different cut-off values. For PCT, the highest sensibility was reached for a cut-off value of 0.10 ng/mL (82.5%; 95% CI: 69.5–90.7%), whereas the highest specificity was reached using a cut-off value of 13.90 ng/mL (98.9%; 95% CI: 97.5–99.5); for PCT-72 h, the maximum sensitivity was 86.2% (95% CI: 69.5–90.7; cut-off value: 0.10 ng/mL) and the maximum specificity was 98.0% (95% CI: 95.8–99.1; cut-off value: 15.60 ng/mL).

Lastly, the highest PPVs were reached using cut-off values of PCT (67.9%, 95% CI: 34.0–89.7) and PCT-72 h (58.8%, 95% CI: 31.5–81.6) of 13.90 ng/mL and 10.40 ng/mL, respectively, while the highest NPVs values were obtained for cut-off values of PCT (66.6%; 95% CI: 60.8–71.9) and PCT-72 h (69.4%; 95% CI: 58.1–78.8) of 0.30 ng/mL and 0.20 ng/mL, respectively.

## 4. Discussion

Bacterial superinfections, including VAP and BSI, are a dangerous complication in COVID-19 patients admitted to the ICU [1,2,3,4,27,28,29,30]. The prompt recognition and appropriate treatment of superinfections are crucial in managing these cases effectively. The diagnosis of superinfections in COVID-19 patients typically involves clinical evaluation, microbiological tests, and imaging studies. In this context, a biomarker such as PCT, a widely used clinical practice to rapidly detect severe bacterial infections in the period before the pandemic, was thought to play a key role in early diagnosis and the modulation of antibiotic therapy even in the superinfected COVID-19 patients [5,6,7,8,9,10,11,12,13,14,15,16,31].

In any case, the SARS-CoV-2 pandemic was a severe test for many of the diagnostic resources designed for contexts other than COVID-19: our results showed that abnormal procalcitonin levels in critically ill COVID-19 patients do not necessarily indicate the presence of a bacterial coinfection or superinfection. In particular, the adjusted sensitivity and specificity for the PCT test in COVID-19 patients were 18.7% and 79.0%, respectively; the PPV was 33.9% and PNV 62.9%. Similarly, also, PCT-72 h model analysis confirmed the limits of PCT as a biomarker of superinfection in SARS-CoV-2-infected patients (35.7% and 69.3%, respectively; PPV: 30.7%; PNV: 70.2%).

Similarly to our experience, a recent review and meta-analysis on the topic that encompassed five large studies involving a total of 2775 patients has shown that the predictive ability of PCT for the diagnosis of coinfections in COVID-19 patients was limited, with an AUC of 0.72, sensitivity of 0.60, and specificity of 0.71 [32]. Additionally, three of the included studies indicated that PCT was an effective tool for ruling out bacterial coinfections, boasting a negative predictive value of over 93% when its concentration was below 0.50 μg/L [33,34,35].

As previously reported, PCT blood levels selectively increase in response to systemic bacterial infections in COVID-19-negative patients and, for this reason, is used as a biomarker for differentiating between bacterial and viral infections. Recently, however, concerns about the appropriateness of PCT in the management of SARS-CoV-2-infected patients arose. In fact, several studies observed that elevated PCT levels may not reflect bacterial coinfections or superinfections in a severe COVID-19 setting [36,37,38]. In this case, the increase of PCT levels, even in the absence of bacterial coinfection, has been hypothesized to be a part of the human immunological response to SARS-CoV-2 infection [39,40]. In particular, a biological model has been proposed in which the increase in PCT production could be related to SARS-CoV-2 ORF6 and NSP1. These SARS-CoV-2 proteins inhibit host STAT1 phosphorylation and increase STAT3-dependent transcriptional pathways. The resulting increase of STAT3 signaling enhances an unexpected PCT production in monocytes in COVID-19 infection not linked to concomitant bacteremia [39].

This hypothesis was indirectly corroborated by a cohort study conducted in the pre-pandemic period: the authors reported that PCT levels increased during pure non-COVID viral infections in correlation with the severity of the disease, and severe respiratory viral infection induces a PCT increase also in the absence of concomitant bacterial pneumonia [11]. Moreover, they demonstrated that PCT synthesis was not suppressed by interferon signaling; PCT concentration was elevated despite bacteriologic sterility and its levels correlated with markers of disease severity in murine and cellular models of influenza infection [11].

In the clinical practice, these pieces of evidence were recently confirmed in a retrospective study involving hospitalized patients with severe COVID-19 and designed to evaluate the effectiveness of PCT in diagnosing respiratory superinfection. The results indicated that PCT levels measured at the time of lower respiratory culture were not able to distinguish patients with a bacterial infection and its diagnostic accuracy was not influenced by factors such as the timing of the procalcitonin measurement, exposure to antibiotics, or treatment with immunomodulatory agents [41].

Our research diverges from several previous reports supporting the utility of PCT and does not endorse the extensive use of this biomarker in the setting of critically ill COVID-19 patients for the management of bacterial superinfections [42,43,44]. The multivariable GEE models conducted on the cohort confirmed that neither PCT nor PCT-72 h were associated with a diagnosis of VAP/BSI.

However, the results obtained from our study may be of support in identifying new PCT operating values that can more precisely support the clinical diagnosis of superinfection even in the setting of critically ill COVID-19 patients. In fact, we observed that the diagnostic accuracy of PCT in predicting an underlying VAP/BSI in COVID-19 was recovered when considering different cut-off values. In particular, the highest sensibility was reached for a cut-off value of 0.10 ng/mL whereas the highest specificity was reached using a cut-off value of 13.90 ng/mL; similarly, for PCT-72 h, the maximum sensitivity available was 86.2% with a cut-off value of 0.10 ng/mL, and the maximum specificity was 98.0% for a cut-off value of 15.60 ng/mL. These new cut-offs should be kept in mind by clinicians when redesigning the risk management of superinfections and for developing new antibiotic stewardship strategies in the context of COVID-19.

Unlike many other studies on the topic, the main strength of the study is that it is based on a homogeneous cohort for clinical severity: the feature significantly reduces the risk of bias, minimizing the effect of this potential confounder. Notwithstanding this, the study has several limitations. First of all, the retrospective nature of the data could reduce the quality of the results and restrict their interpretation. Secondarily, the study was restricted to critically ill patients managed in ICUs and supported with mechanical ventilation. Moreover, the design of the study evaluates the PCT in relation to chronologically synchronous superinfections marked by the simultaneous microbiological isolation of the causal bacterial pathogen in blood or in BAL cultures. Consequently, the results obtained can be related to the diagnostic accuracy of microbiological cultures. For this reason, we performed an adjusted analysis. Moreover, we underline that the limitation of poor blood culture sensitivity could also be overcome by increasing the number of blood culture sets taken, ensuring a blood culture sensitivity of at least 95%. [25] Another important aspect to consider is that our study was conducted in the early stages of the pandemic, before the emergence of the Omicron variant and the availability of vaccines, both of which have led to a change in the severity of the disease towards milder cases. Finally, in this study, we evaluated PCT as a “static” value; in any case, it is used also as a “dynamic” marker and its variation could possibly better describe a bacterial superinfection.

## 5. Conclusions

The validation of diagnostic tools in contexts other than those in which they were previously tested and approved is one of the most significant concerns in clinical practice. Monitoring procalcitonin levels could help healthcare providers to assess the likelihood of a bacterial infection and guide decisions regarding the use of antibiotics in the pre-pandemic period, but it can mislead clinicians in the setting of severe COVID-19. It is worth noting that procalcitonin levels alone can be not sufficient for use in differentiating between viral SARS-CoV-2 infection and bacterial infections. Additional clinical evaluations, including other laboratory tests and imaging studies, are required in order to make an accurate diagnosis of superinfection in critically ill COVID-19 patients.

## Figures and Tables

**Table 1:** Characteristics of the COVID-19 patients who recovered in the ICU from March 2020 to February 2021.

		COVID-19 Patients *n* (%)
Patients		184
Gender		
	Female	48 (26.1)
	Male	136 (73.9)
Age, years		
	Median (IQR)	66 (55.5, 73)
SAPS II (*n* = 162)		
	Median (IQR)	35 (27, 43)
Comorbidities (yes)		146 (79.4)
	Diabetes mellitus (yes)	40 (21.7)
	Obesity (yes)	22 (12.0)
	Hypertension (yes)	86 (46.7)
	Cardiopathy (yes)	35 (19.0)
	Renal failure (yes)	12 (6.5)
	COPD (yes)	26 (14.1)
	Hepatopathy (yes)	4 (2.2)
	Neurological disorders (yes)	28 (15.2)
	Other disorders (yes)	63 (34.3)
Outcome		
	Discharge	115 (62.5)
	Death	69 (37.5)

SAPS: Simplified Acute Physiology Score.

**Table 2:** Multivariable models predicting VAP/BSI in COVID-19 patients hospitalized in the intensive care unit from March 2020 to February 2021.

	Model 1 ^a^ (*n* = 162, Observations = 1702)			Model 2 ^b^ (*n* = 162, Observations = 1810)		
	aOR	95% CI	*p*-Value	aOR	95% CI	*p*-Value
Positive PCT (yes)	1.13	0.51–2.52	0.764	--	--	--
Positive PCT-72 h (yes)	--	--	--	1.32	0.66–2.64	0.427
Age in years	1.02	1.00–1.05	0.099	1.02	1.00–1.05	0.099
Gender (male)	1.00	0.57–1.05	0.993	0.98	0.58–1.67	0.939
SAPS II	0.98	0.95–1.01	0.202	0.98	0.95–1.01	0.129
Comorbidity (yes)	1.13	0.55–2.39	0.623	1.25	0.62–2.51	0.536

^a^ Multivariable generalized estimating equation regression model (logit link, binomial error structure, exchangeable correlation structure) ^b^ Multivariable generalized estimating equation regression model (logit link, binomial error structure, exchangeable correlation structure) considering the PCT as positive 24 h before and after the PCR positive test. aOR: adjusted odds ratio; CI: confidence interval; PCT: procalcitonin; SAPS: Simplified Acute Physiology Score.

## Supplementary Materials

The following supporting information can be downloaded at: https://www.mdpi.com/article/10.3390/jcm12196171/s1, Table S1: The diagnostic accuracy of PCT and PCT-72 h values in predicting an underlying VAP/BSI, considering different cut-off values.
