# Supplementary material for: Reduced Reliability of Procalcitonin (PCT) as a Biomarker of Bacterial Superinfection: Concerns about PCT-Driven Antibiotic Stewardship in Critically Ill COVID-19 Patients—Results from a Retrospective Observational Study in Intensive Care Units
Source: J Clin Med. 2023 Sep 24;12(19):6171. doi: 10.3390/jcm12196171 (PMC10573961; doi:10.3390/jcm12196171)
Supplement: Supplementary file 1 [file jcm-12-06171-s001.zip › jcm-2605959-supplementary.pdf]

Supplementary material

Table S1: the diagnostic accuracy of PCT and PCT-72h values in predicting an underlying a VAP/BSI considering different cut-off values.

PCT	Cut-off	Prevalenxce	Sensibility	Sens_low	Sens_high	Specificity	Spec_low	Spec_high	VPP	VPP_low	VPP_high	VPN	VPN_low	VPN_high
PCT 72h	0.10	0.364130	0.861866	0.755204	0.926572	0.120875	0.087235	0.165142	0.359552	0.333617	0.386335	0.604444	.42710011	.75799632
PCT	0.10	0.364130	0.825281	0.694888	0.907376	0.162636	0.122995	0.211966	0.360772	0.330972	0.391683	0.619120	0.465547	0.752066
PCT 72h	0.20	0.364130	0.748324	0.617756	0.845450	0.326488	0.272819	0.385124	0.388849	0.349922	0.429248	0.693755	.58062878	.78753146
PCT	0.20	0.364130	0.620808	0.465795	0.754542	0.414767	0.357877	0.474024	0.377900	0.326730	0.431943	0.656369	0.569786	0.733671
PCT 72h	0.30	0.364130	0.557703	0.417405	0.689359	0.555677	0.494528	0.615185	0.418191	0.357218	0.481770	0.686905	.61427095	.75139649
PCT	0.30	0.364130	0.436442	0.295397	0.588576	0.642698	0.584764	0.696741	0.411587	0.335173	0.492517	0.665719	0.607668	0.719150
PCT 72h	0.40	0.364130	0.442601	0.310231	0.583660	0.687513	0.626895	0.742330	0.447847	0.369522	0.528848	0.682932	.62556679	.73522924
PCT	0.40	0.364130	0.285829	0.171292	0.436602	0.753018	0.697977	0.800890	0.398575	0.296106	0.510773	0.648043	0.605754	0.688130
PCT 72h	0.50	0.364130	0.308416	0.197196	0.447405	0.770468	0.712547	0.819672	0.434853	0.333099	0.542411	0.660493	.6165365	.70184838
PCT	0.50	0.364130	0.191842	0.101449	0.332934	0.815919	0.763553	0.858832	0.373745	0.248797	0.518159	0.638080	0.605263	0.669657
PCT 72h	0.60	0.364130	0.275107	0.168524	0.415421	0.807904	0.752319	0.853445	0.450583	0.339091	0.567269	0.660585	.62014151	.69881311
PCT	0.60	0.364130	0.191842	0.101449	0.332934	0.847983	0.799135	0.886638	0.419506	0.285823	0.566149	0.646933	0.614519	0.678049
PCT 72h	0.70	0.364130	0.275107	0.168524	0.415421	0.824945	0.770648	0.868580	0.473669	0.359941	0.590197	0.665249	.62508309	.70315524
PCT	0.70	0.364130	0.169319	0.085783	0.306894	0.862904	0.815594	0.899572	0.414262	0.272144	0.572246	0.644635	0.614549	0.673620
PCT 72h	0.80	0.364130	0.275107	0.168524	0.415421	0.835713	0.782627	0.877858	0.489518	0.374491	0.605665	0.668131	.62813987	.705835
PCT	0.80	0.364130	0.169319	0.085783	0.306894	0.869837	0.823275	0.905539	0.426907	0.282410	0.585058	0.646466	0.616461	0.675360
PCT 72h	0.90	0.364130	0.263553	0.157282	0.406953	0.842562	0.790341	0.883690	0.489437	0.371156	0.608912	0.666432	.62753625	.70318705
PCT	0.90	0.364130	0.154252	0.073714	0.294780	0.879083	0.834481	0.912920	0.422138	0.271266	0.589091	0.644772	0.616352	0.672207
PCT 72h	1.00	0.364130	0.263553	0.157282	0.406953	0.848306	0.797567	0.888111	0.498726	0.379745	0.617853	0.667941	.62913473	.70459244
PCT	1.00	0.364130	0.154252	0.073714	0.294780	0.885746	0.843182	0.917883	0.436024	0.282458	0.602925	0.646500	0.618154	0.673852
PCT 72h	1.10	0.364130	0.241387	0.140376	0.382721	0.859907	0.810687	0.897941	0.496653	0.370986	0.622744	0.664366	.62764582	.69920108
PCT	1.10	0.364130	0.131148	0.058766	0.267359	0.895431	0.854605	0.925788	0.417997	0.255063	0.601035	0.642817	0.616935	0.667893
PCT 72h	1.20	0.364130	0.219582	0.124092	0.358480	0.866362	0.818617	0.903029	0.484783	0.352693	0.619035	0.659700	.6249904	.69277532
PCT	1.20	0.364130	0.131148	0.058766	0.267359	0.901893	0.862470	0.930920	0.433591	0.267191	0.616445	0.644467	0.618651	0.669469
PCT 72h	1.30	0.364130	0.198136	0.108434	0.334226	0.869323	0.821949	0.905542	0.464746	0.326757	0.608351	0.654360	.62163144	.68568807
PCT	1.30	0.364130	0.131148	0.058766	0.267359	0.907002	0.868748	0.934941	0.446769	0.277629	0.629200	0.645760	0.619997	0.670704
PCT 72h	1.40	0.364130	0.198136	0.108434	0.334226	0.873622	0.827089	0.909010	0.473075	0.334049	0.616405	0.655474	.62280105	.68674085
PCT	1.40	0.364130	0.131148	0.058766	0.267359	0.913552	0.877898	0.939513	0.464885	0.292267	0.646346	0.647404	0.621709	0.672274
PCT 72h	1.50	0.364130	0.198136	0.108434	0.334226	0.878845	0.833908	0.912893	0.483606	0.343360	0.626483	0.656819	.62421247	.68801055
PCT	1.50	0.364130	0.131148	0.058766	0.267359	0.918730	0.884737	0.943340	0.480277	0.304976	0.660573	0.648693	0.623052	0.673505
PCT 72h	1.60	0.364130	0.198136	0.108434	0.334226	0.882479	0.837763	0.916104	0.491215	0.350151	0.633692	0.657749	.62518838	.68888799
PCT	1.60	0.364130	0.131148	0.058766	0.267359	0.921721	0.888051	0.945881	0.489642	0.312835	0.669080	0.649434	0.623823	0.674212
PCT 72h	1.70	0.364130	0.198136	0.108434	0.334226	0.888330	0.845389	0.920467	0.503981	0.361668	0.645653	0.659235	.62674895	.69029033
PCT	1.70	0.364130	0.131148	0.058766	0.267359	0.927605	0.895288	0.950500	0.509178	0.329543	0.686474	0.650881	0.625331	0.675593
PCT 72h	1.80	0.364130	0.180833	0.095418	0.316000	0.888576	0.845757	0.920623	0.481696	0.334135	0.632520	0.654485	.62360468	.68411565
PCT	1.80	0.364130	0.131148	0.058766	0.267359	0.928232	0.896293	0.950874	0.511351	0.331428	0.688380	0.651035	0.625491	0.675740
PCT 72h	1.90	0.364130	0.163026	0.082436	0.296909	0.896619	0.854835	0.927398	0.474525	0.319194	0.634949	0.651655	.62256945	.67965178
PCT	1.90	0.364130	0.131148	0.058766	0.267359	0.933428	0.902230	0.955165	0.530104	0.347929	0.704596	0.652302	0.626811	0.676949
PCT 72h	2.00	0.364130	0.150739	0.072038	0.288674	0.904415	0.865134	0.933139	0.474536	0.312485	0.642134	0.650310	.62252053	.67711534
PCT	2.00	0.364130	0.131148	0.058766	0.267359	0.939028	0.909767	0.959225	0.551919	0.367656	0.722949	0.653657	0.628224	0.678242
PCT 72h	2.10	0.364130	0.128208	0.057460	0.261864	0.909643	0.871619	0.937216	0.448287	0.276892	0.632915	0.645652	.62022578	.6702816

PCT	2.10	0.364130	0.108408	0.044777	0.239765	0.941375	0.913018	0.960883	0.514311	0.315354	0.708833	0.648355	0.625389	0.670654
PCT 72h	2.20	0.364130	0.128208	0.057460	0.261864	0.910975	0.873519	0.938124	0.451961	0.279826	0.636413	0.645987	.62057401	.67060128
PCT	2.20	0.364130	0.108408	0.044777	0.239765	0.942946	0.915379	0.961906	0.521092	0.321114	0.714531	0.648735	0.625784	0.671017
PCT 72h	2.30	0.364130	0.128208	0.057460	0.261864	0.911185	0.873838	0.938258	0.452549	0.280296	0.636970	0.646040	.62062906	.67065183
PCT	2.30	0.364130	0.108408	0.044777	0.239765	0.943591	0.916454	0.962276	0.523930	0.323541	0.716898	0.648890	0.625946	0.671167
PCT 72h	2.40	0.364130	0.128208	0.057460	0.261864	0.911934	0.874694	0.938879	0.454646	0.281979	0.638956	0.646228	.62082449	.67083121
PCT	2.40	0.364130	0.108408	0.044777	0.239765	0.944218	0.917197	0.962779	0.526718	0.325937	0.719215	0.649042	0.626104	0.671312
PCT 72h	2.50	0.364130	0.128208	0.057460	0.261864	0.913197	0.876339	0.939823	0.458231	0.284865	0.642337	0.646544	.62115382	.67113349
PCT	2.50	0.364130	0.108408	0.044777	0.239765	0.945149	0.918460	0.963450	0.530913	0.329559	0.722681	0.649266	0.626337	0.671527
PCT 72h	2.60	0.364130	0.128208	0.057460	0.261864	0.916231	0.879520	0.942488	0.467076	0.292044	0.650606	0.647302	.62194255	.67185731
PCT	2.60	0.364130	0.108408	0.044777	0.239765	0.946922	0.920407	0.964941	0.539087	0.336683	0.729374	0.649693	0.626781	0.671935
PCT 72h	2.70	0.364130	0.128208	0.057460	0.261864	0.920189	0.883998	0.945781	0.479139	0.301971	0.661715	0.648285	.6229663	.67279655
PCT	2.70	0.364130	0.086028	0.031874	0.212039	0.949259	0.922898	0.966930	0.492615	0.274211	0.713874	0.644595	0.624315	0.664368
PCT 72h	2.80	0.364130	0.128208	0.057460	0.261864	0.920782	0.884701	0.946257	0.481000	0.303516	0.663413	0.648432	.62311922	.67293681
PCT	2.80	0.364130	0.086028	0.031874	0.212039	0.949947	0.923671	0.967496	0.496027	0.276874	0.716718	0.644761	0.624487	0.664528
PCT 72h	2.90	0.364130	0.128208	0.057460	0.261864	0.924175	0.887957	0.949353	0.491937	0.312676	0.673296	0.649270	.62399194	.67373721
PCT	2.90	0.364130	0.086028	0.031874	0.212039	0.951028	0.924504	0.968551	0.501489	0.281168	0.721234	0.645021	0.624758	0.664778
PCT 72h	3.00	0.364130	0.128208	0.057460	0.261864	0.926297	0.891388	0.950608	0.499034	0.318691	0.679628	0.649792	.6245358	.67423589
PCT	3.00	0.364130	0.086028	0.031874	0.212039	0.952140	0.925911	0.969391	0.507229	0.285721	0.725935	0.645289	0.625035	0.665035
PCT 72h	3.10	0.364130	0.106016	0.043805	0.234877	0.927607	0.893255	0.951504	0.456112	0.266152	0.659761	0.644374	.62154614	.66656433
PCT	3.10	0.364130	0.086028	0.031874	0.212039	0.953380	0.927687	0.970237	0.513787	0.290975	0.731250	0.645587	0.625344	0.665321
PCT 72h	3.20	0.364130	0.106016	0.043805	0.234877	0.931231	0.897460	0.954445	0.468880	0.276115	0.671401	0.645267	.62247386	.66742021
PCT	3.20	0.364130	0.086028	0.031874	0.212039	0.955457	0.930150	0.971872	0.525162	0.300225	0.740331	0.646084	0.625860	0.665799
PCT 72h	3.30	0.364130	0.106016	0.043805	0.234877	0.932244	0.898342	0.955402	0.472577	0.279035	0.674729	0.645516	.62273237	.66765867
PCT	3.30	0.364130	0.086028	0.031874	0.212039	0.955951	0.930505	0.972356	0.527942	0.302513	0.742525	0.646202	0.625983	0.665912
PCT 72h	3.40	0.364130	0.106016	0.043805	0.234877	0.932947	0.899097	0.955997	0.475177	0.281097	0.677056	0.645689	.62291148	.66782387
PCT	3.40	0.364130	0.086028	0.031874	0.212039	0.956635	0.931159	0.972957	0.531842	0.305740	0.745584	0.646366	0.626152	0.666070
PCT 72h	3.50	0.364130	0.106016	0.043805	0.234877	0.935791	0.902924	0.958047	0.485993	0.289761	0.686641	0.646385	.62363449	.66849068
PCT	3.50	0.364130	0.086028	0.031874	0.212039	0.958860	0.933725	0.974721	0.544935	0.316727	0.755711	0.646897	0.626703	0.666580
PCT 72h	3.60	0.364130	0.106016	0.043805	0.234877	0.936166	0.903453	0.958307	0.487460	0.290946	0.687928	0.646476	.62372984	.6685786
PCT	3.60	0.364130	0.086028	0.031874	0.212039	0.959259	0.934210	0.975026	0.547349	0.318780	0.757554	0.646992	0.626802	0.666671
PCT 72h	3.70	0.364130	0.106016	0.043805	0.234877	0.938261	0.905878	0.959995	0.495799	0.297735	0.695191	0.646987	.62426044	.66906787
PCT	3.70	0.364130	0.086028	0.031874	0.212039	0.960806	0.936434	0.976072	0.556922	0.327002	0.764792	0.647360	0.627184	0.667024
PCT 72h	3.80	0.364130	0.106016	0.043805	0.234877	0.940783	0.908688	0.962068	0.506226	0.306340	0.704142	0.647600	.62489731	.66965503
PCT	3.80	0.364130	0.086028	0.031874	0.212039	0.962277	0.938185	0.977208	0.566339	0.335223	0.771803	0.647709	0.627546	0.667359
PCT 72h	3.90	0.364130	0.106016	0.043805	0.234877	0.944305	0.912946	0.964803	0.521539	0.319221	0.717029	0.648452	.6257828	.67047125
PCT	3.90	0.364130	0.086028	0.031874	0.212039	0.963803	0.939656	0.978508	0.576449	0.344197	0.779211	0.648070	0.627921	0.667706
PCT 72h	4.00	0.364130	0.106016	0.043805	0.234877	0.945390	0.914046	0.965732	0.526447	0.323412	0.721097	0.648714	.62605489	.67072201
PCT	4.00	0.364130	0.064004	0.020265	0.184384	0.964723	0.940684	0.979235	0.509557	0.255279	0.758984	0.642840	0.625556	0.659759
PCT 72h	4.10	0.364130	0.106016	0.043805	0.234877	0.946650	0.915157	0.966876	0.532262	0.328418	0.725877	0.649017	.62637027	.67101265
PCT	4.10	0.364130	0.064004	0.020265	0.184384	0.965373	0.941152	0.979839	0.514206	0.258733	0.762466	0.642995	0.625717	0.659908
PCT 72h	4.20	0.364130	0.084160	0.031197	0.207754	0.947855	0.917332	0.967508	0.480312	0.262525	0.705848	0.643787	.6237253	.66335727
PCT	4.20	0.364130	0.064004	0.020265	0.184384	0.966621	0.943451	0.980493	0.523367	0.265627	0.769234	0.643291	0.626023	0.660194
PCT 72h	4.30	0.364130	0.084160	0.031197	0.207754	0.949698	0.919262	0.969047	0.489300	0.269394	0.713427	0.644233	.62418713	.66378523
PCT	4.30	0.364130	0.064004	0.020265	0.184384	0.967550	0.944516	0.981212	0.530406	0.271007	0.774355	0.643511	0.626252	0.660406
PCT 72h	4.40	0.364130	0.084160	0.031197	0.207754	0.949698	0.919262	0.969047	0.489300	0.269394	0.713427	0.644233	.62418713	.66378523
PCT	4.40	0.364130	0.064004	0.020265	0.184384	0.967550	0.944516	0.981212	0.530406	0.271007	0.774355	0.643511	0.626252	0.660406

PCT 72h	4.50	0.364130	0.084160	0.031197	0.207754	0.949698	0.919262	0.969047	0.489300	0.269394	0.713427	0.644233	.62418713	.66378523
PCT	4.50	0.364130	0.064004	0.020265	0.184384	0.967976	0.945211	0.981468	0.533697	0.273547	0.776726	0.643612	0.626356	0.660503
PCT 72h	4.60	0.364130	0.084160	0.031197	0.207754	0.950362	0.920315	0.969456	0.492622	0.271959	0.716198	0.644393	.62435329	.66393919
PCT	4.60	0.364130	0.064004	0.020265	0.184384	0.968364	0.945809	0.981713	0.536728	0.275900	0.778895	0.643704	0.626452	0.660592
PCT 72h	4.70	0.364130	0.084160	0.031197	0.207754	0.950906	0.920998	0.969862	0.495376	0.274095	0.718483	0.644524	.62448922	.66406513
PCT	4.70	0.364130	0.064004	0.020265	0.184384	0.968960	0.946584	0.982140	0.541452	0.279596	0.782254	0.643845	0.626598	0.660728
PCT 72h	4.80	0.364130	0.084160	0.031197	0.207754	0.951227	0.921493	0.970066	0.497017	0.275373	0.719840	0.644601	.62456944	.66413947
PCT	4.80	0.364130	0.064004	0.020265	0.184384	0.969308	0.947081	0.982373	0.544253	0.281803	0.784230	0.643928	0.626683	0.660807
PCT 72h	4.90	0.364130	0.084160	0.031197	0.207754	0.952542	0.922638	0.971247	0.503846	0.280727	0.725444	0.644918	.62489741	.66444333
PCT	4.90	0.364130	0.064004	0.020265	0.184384	0.969748	0.947323	0.982800	0.547832	0.284641	0.786740	0.644032	0.626791	0.660907
PCT 72h	5.00	0.364130	0.084160	0.031197	0.207754	0.955620	0.925629	0.973858	0.520600	0.294122	0.738916	0.645656	.6256633	.66515286
PCT	5.00	0.364130	0.064004	0.020265	0.184384	0.971510	0.948888	0.984286	0.562651	0.296602	0.796958	0.644448	0.627222	0.661308
PCT 72h	5.10	0.364130	0.084160	0.031197	0.207754	0.955620	0.925629	0.973858	0.520600	0.294122	0.738916	0.645656	.6256633	.66515286
PCT	5.10	0.364130	0.064004	0.020265	0.184384	0.971510	0.948888	0.984286	0.562651	0.296602	0.796958	0.644448	0.627222	0.661308
PCT 72h	5.20	0.364130	0.084160	0.031197	0.207754	0.955620	0.925629	0.973858	0.520600	0.294122	0.738916	0.645656	.6256633	.66515286
PCT	5.20	0.364130	0.064004	0.020265	0.184384	0.971510	0.948888	0.984286	0.562651	0.296602	0.796958	0.644448	0.627222	0.661308
PCT 72h	5.30	0.364130	0.084160	0.031197	0.207754	0.955620	0.925629	0.973858	0.520600	0.294122	0.738916	0.645656	.6256633	.66515286
PCT	5.30	0.364130	0.064004	0.020265	0.184384	0.971510	0.948888	0.984286	0.562651	0.296602	0.796958	0.644448	0.627222	0.661308
PCT 72h	5.40	0.364130	0.084160	0.031197	0.207754	0.956139	0.926186	0.974275	0.523534	0.296506	0.741236	0.645780	.62579209	.66527216
PCT	5.40	0.364130	0.064004	0.020265	0.184384	0.971677	0.948998	0.984437	0.564093	0.297785	0.797937	0.644487	0.627262	0.661346
PCT 72h	5.50	0.364130	0.084160	0.031197	0.207754	0.956139	0.926186	0.974275	0.523534	0.296506	0.741236	0.645780	.62579209	.66527216
PCT	5.50	0.364130	0.064004	0.020265	0.184384	0.971677	0.948998	0.984437	0.564093	0.297785	0.797937	0.644487	0.627262	0.661346
PCT 72h	5.60	0.364130	0.084160	0.031197	0.207754	0.956423	0.926621	0.974454	0.525154	0.297827	0.742512	0.645848	.62586252	.6653374
PCT	5.60	0.364130	0.064004	0.020265	0.184384	0.971955	0.949332	0.984640	0.566514	0.299777	0.799575	0.644553	0.627330	0.661409
PCT 72h	5.70	0.364130	0.084160	0.031197	0.207754	0.956423	0.926621	0.974454	0.525154	0.297827	0.742512	0.645848	.62586252	.6653374
PCT	5.70	0.364130	0.064004	0.020265	0.184384	0.971955	0.949332	0.984640	0.566514	0.299777	0.799575	0.644553	0.627330	0.661409
PCT 72h	5.80	0.364130	0.084160	0.031197	0.207754	0.956423	0.926621	0.974454	0.525154	0.297827	0.742512	0.645848	.62586252	.6653374
PCT	5.80	0.364130	0.064004	0.020265	0.184384	0.971955	0.949332	0.984640	0.566514	0.299777	0.799575	0.644553	0.627330	0.661409
PCT 72h	5.90	0.364130	0.084160	0.031197	0.207754	0.956423	0.926621	0.974454	0.525154	0.297827	0.742512	0.645848	.62586252	.6653374
PCT	5.90	0.364130	0.064004	0.020265	0.184384	0.971955	0.949332	0.984640	0.566514	0.299777	0.799575	0.644553	0.627330	0.661409
PCT 72h	6.00	0.364130	0.084160	0.031197	0.207754	0.956423	0.926621	0.974454	0.525154	0.297827	0.742512	0.645848	.62586252	.6653374
PCT	6.00	0.364130	0.064004	0.020265	0.184384	0.971955	0.949332	0.984640	0.566514	0.299777	0.799575	0.644553	0.627330	0.661409
PCT 72h	6.10	0.364130	0.084160	0.031197	0.207754	0.957835	0.928375	0.975498	0.533365	0.304581	0.748926	0.646186	.62621262	.66566168
PCT	6.10	0.364130	0.064004	0.020265	0.184384	0.972338	0.949551	0.984995	0.569890	0.302573	0.801847	0.644643	0.627424	0.661496
PCT 72h	6.20	0.364130	0.084160	0.031197	0.207754	0.957835	0.928375	0.975498	0.533365	0.304581	0.748926	0.646186	.62621262	.66566168
PCT	6.20	0.364130	0.064004	0.020265	0.184384	0.973497	0.952131	0.985472	0.580351	0.311354	0.808799	0.644916	0.627706	0.661759
PCT 72h	6.30	0.364130	0.084160	0.031197	0.207754	0.957835	0.928375	0.975498	0.533365	0.304581	0.748926	0.646186	.62621262	.66566168
PCT	6.30	0.364130	0.064004	0.020265	0.184384	0.973497	0.952131	0.985472	0.580351	0.311354	0.808799	0.644916	0.627706	0.661759
PCT 72h	6.40	0.364130	0.084160	0.031197	0.207754	0.958083	0.928677	0.975683	0.534830	0.305796	0.750061	0.646245	.62627388	.66571842
PCT	6.40	0.364130	0.064004	0.020265	0.184384	0.973789	0.952493	0.985682	0.583045	0.313645	0.810568	0.644985	0.627777	0.661825
PCT 72h	6.50	0.364130	0.084160	0.031197	0.207754	0.958083	0.928677	0.975683	0.534830	0.305796	0.750061	0.646245	.62627388	.66571842
PCT	6.50	0.364130	0.064004	0.020265	0.184384	0.973789	0.952493	0.985682	0.583045	0.313645	0.810568	0.644985	0.627777	0.661825
PCT 72h	6.60	0.364130	0.084160	0.031197	0.207754	0.958083	0.928677	0.975683	0.534830	0.305796	0.750061	0.646245	.62627388	.66571842
PCT	6.60	0.364130	0.064004	0.020265	0.184384	0.973789	0.952493	0.985682	0.583045	0.313645	0.810568	0.644985	0.627777	0.661825
PCT 72h	6.70	0.364130	0.084160	0.031197	0.207754	0.958083	0.928677	0.975683	0.534830	0.305796	0.750061	0.646245	.62627388	.66571842
PCT	6.70	0.364130	0.064004	0.020265	0.184384	0.974905	0.955279	0.986044	0.593585	0.322730	0.817405	0.645247	0.628049	0.662077
PCT 72h	6.80	0.364130	0.084160	0.031197	0.207754	0.958083	0.928677	0.975683	0.534830	0.305796	0.750061	0.646245	.62627388	.66571842

PCT	6.80	0.364130	0.064004	0.020265	0.184384	0.974905	0.955279	0.986044	0.593585	0.322730	0.817405	0.645247	0.628049	0.662077
PCT 72h	6.90	0.364130	0.084160	0.031197	0.207754	0.958416	0.929241	0.975874	0.536816	0.307448	0.751595	0.646324	.62635639	.66579484
PCT	6.90	0.364130	0.064004	0.020265	0.184384	0.975509	0.956370	0.986372	0.599445	0.327866	0.821151	0.645388	0.628196	0.662214
PCT 72h	7.00	0.364130	0.084160	0.031197	0.207754	0.958416	0.929241	0.975874	0.536816	0.307448	0.751595	0.646324	.62635639	.66579484
PCT	7.00	0.364130	0.064004	0.020265	0.184384	0.975509	0.956370	0.986372	0.599445	0.327866	0.821151	0.645388	0.628196	0.662214
PCT 72h	7.10	0.364130	0.084160	0.031197	0.207754	0.958416	0.929241	0.975874	0.536816	0.307448	0.751595	0.646324	.62635639	.66579484
PCT	7.10	0.364130	0.064004	0.020265	0.184384	0.975509	0.956370	0.986372	0.599445	0.327866	0.821151	0.645388	0.628196	0.662214
PCT 72h	7.20	0.364130	0.084160	0.031197	0.207754	0.958416	0.929241	0.975874	0.536816	0.307448	0.751595	0.646324	.62635639	.66579484
PCT	7.20	0.364130	0.064004	0.020265	0.184384	0.975509	0.956370	0.986372	0.599445	0.327866	0.821151	0.645388	0.628196	0.662214
PCT 72h	7.30	0.364130	0.084160	0.031197	0.207754	0.958416	0.929241	0.975874	0.536816	0.307448	0.751595	0.646324	.62635639	.66579484
PCT	7.30	0.364130	0.064004	0.020265	0.184384	0.975509	0.956370	0.986372	0.599445	0.327866	0.821151	0.645388	0.628196	0.662214
PCT 72h	7.40	0.364130	0.084160	0.031197	0.207754	0.958416	0.929241	0.975874	0.536816	0.307448	0.751595	0.646324	.62635639	.66579484
PCT	7.40	0.364130	0.042330	0.010325	0.157724	0.975509	0.956370	0.986372	0.497423	0.199791	0.796893	0.640132	0.626213	0.653819
PCT 72h	7.50	0.364130	0.084160	0.031197	0.207754	0.959386	0.931322	0.976275	0.542678	0.312356	0.756094	0.646556	.62659615	.6660169
PCT	7.50	0.364130	0.042330	0.010325	0.157724	0.976556	0.958708	0.986796	0.508349	0.206636	0.804101	0.640379	0.626469	0.654057
PCT 72h	7.60	0.364130	0.084160	0.031197	0.207754	0.959386	0.931322	0.976275	0.542678	0.312356	0.756094	0.646556	.62659615	.6660169
PCT	7.60	0.364130	0.042330	0.010325	0.157724	0.976556	0.958708	0.986796	0.508349	0.206636	0.804101	0.640379	0.626469	0.654057
PCT 72h	7.70	0.364130	0.084160	0.031197	0.207754	0.959386	0.931322	0.976275	0.542678	0.312356	0.756094	0.646556	.62659615	.6660169
PCT	7.70	0.364130	0.042330	0.010325	0.157724	0.976556	0.958708	0.986796	0.508349	0.206636	0.804101	0.640379	0.626469	0.654057
PCT 72h	7.80	0.364130	0.084160	0.031197	0.207754	0.960431	0.933489	0.976731	0.549139	0.317822	0.761002	0.646804	.62685411	.6662558
PCT	7.80	0.364130	0.042330	0.010325	0.157724	0.977604	0.960788	0.987304	0.519776	0.213972	0.811447	0.640626	0.626724	0.654296
PCT 72h	7.90	0.364130	0.084160	0.031197	0.207754	0.960431	0.933489	0.976731	0.549139	0.317822	0.761002	0.646804	.62685411	.6662558
PCT	7.90	0.364130	0.042330	0.010325	0.157724	0.978062	0.961390	0.987628	0.524926	0.217340	0.814695	0.640734	0.626836	0.654400
PCT 72h	8.00	0.364130	0.084160	0.031197	0.207754	0.960431	0.933489	0.976731	0.549139	0.317822	0.761002	0.646804	.62685411	.6662558
PCT	8.00	0.364130	0.042330	0.010325	0.157724	0.978062	0.961390	0.987628	0.524926	0.217340	0.814695	0.640734	0.626836	0.654400
PCT 72h	8.10	0.364130	0.084160	0.031197	0.207754	0.960431	0.933489	0.976731	0.549139	0.317822	0.761002	0.646804	.62685411	.6662558
PCT	8.10	0.364130	0.042330	0.010325	0.157724	0.978062	0.961390	0.987628	0.524926	0.217340	0.814695	0.640734	0.626836	0.654400
PCT 72h	8.20	0.364130	0.084160	0.031197	0.207754	0.960431	0.933489	0.976731	0.549139	0.317822	0.761002	0.646804	.62685411	.6662558
PCT	8.20	0.364130	0.042330	0.010325	0.157724	0.978062	0.961390	0.987628	0.524926	0.217340	0.814695	0.640734	0.626836	0.654400
PCT 72h	8.30	0.364130	0.084160	0.031197	0.207754	0.961246	0.934313	0.977403	0.554288	0.322223	0.764875	0.646998	.62705516	.66644199
PCT	8.30	0.364130	0.042330	0.010325	0.157724	0.978604	0.961824	0.988099	0.531157	0.221467	0.818574	0.640861	0.626968	0.654522
PCT 72h	8.40	0.364130	0.084160	0.031197	0.207754	0.961246	0.934313	0.977403	0.554288	0.322223	0.764875	0.646998	.62705516	.66644199
PCT	8.40	0.364130	0.042330	0.010325	0.157724	0.978604	0.961824	0.988099	0.531157	0.221467	0.818574	0.640861	0.626968	0.654522
PCT 72h	8.50	0.364130	0.084160	0.031197	0.207754	0.963400	0.936605	0.979122	0.568367	0.334455	0.775300	0.647509	.62758524	.66693285
PCT	8.50	0.364130	0.042330	0.010325	0.157724	0.979614	0.962783	0.988921	0.543178	0.229595	0.825906	0.641099	0.627214	0.654751
PCT 72h	8.60	0.364130	0.084160	0.031197	0.207754	0.963400	0.936605	0.979122	0.568367	0.334455	0.775300	0.647509	.62758524	.66693285
PCT	8.60	0.364130	0.042330	0.010325	0.157724	0.979614	0.962783	0.988921	0.543178	0.229595	0.825906	0.641099	0.627214	0.654751
PCT 72h	8.70	0.364130	0.084160	0.031197	0.207754	0.964336	0.938503	0.979554	0.574711	0.340066	0.779920	0.647731	.62781514	.66714573
PCT	8.70	0.364130	0.042330	0.010325	0.157724	0.980581	0.964703	0.989394	0.555207	0.237956	0.833048	0.641326	0.627448	0.654970
PCT 72h	8.80	0.364130	0.084160	0.031197	0.207754	0.964336	0.938503	0.979554	0.574711	0.340066	0.779920	0.647731	.62781514	.66714573
PCT	8.80	0.364130	0.042330	0.010325	0.157724	0.980581	0.964703	0.989394	0.555207	0.237956	0.833048	0.641326	0.627448	0.654970
PCT 72h	8.90	0.364130	0.084160	0.031197	0.207754	0.964336	0.938503	0.979554	0.574711	0.340066	0.779920	0.647731	.62781514	.66714573
PCT	8.90	0.364130	0.042330	0.010325	0.157724	0.980581	0.964703	0.989394	0.555207	0.237956	0.833048	0.641326	0.627448	0.654970
PCT 72h	9.00	0.364130	0.084160	0.031197	0.207754	0.964840	0.938737	0.980057	0.578185	0.343164	0.782429	0.647850	.62793876	.66726019
PCT	9.00	0.364130	0.042330	0.010325	0.157724	0.980729	0.964669	0.989567	0.557097	0.239291	0.834153	0.641360	0.627484	0.655004
PCT 72h	9.10	0.364130	0.084160	0.031197	0.207754	0.964840	0.938737	0.980057	0.578185	0.343164	0.782429	0.647850	.62793876	.66726019
PCT	9.10	0.364130	0.042330	0.010325	0.157724	0.980729	0.964669	0.989567	0.557097	0.239291	0.834153	0.641360	0.627484	0.655004

PCT 72h	9.20	0.364130	0.084160	0.031197	0.207754	0.964840	0.938737	0.980057	0.578185	0.343164	0.782429	0.647850	.62793876	.66726019
PCT	9.20	0.364130	0.042330	0.010325	0.157724	0.980729	0.964669	0.989567	0.557097	0.239291	0.834153	0.641360	0.627484	0.655004
PCT 72h	9.30	0.364130	0.084160	0.031197	0.207754	0.964840	0.938737	0.980057	0.578185	0.343164	0.782429	0.647850	.62793876	.66726019
PCT	9.30	0.364130	0.042330	0.010325	0.157724	0.980729	0.964669	0.989567	0.557097	0.239291	0.834153	0.641360	0.627484	0.655004
PCT 72h	9.40	0.364130	0.084160	0.031197	0.207754	0.965361	0.939219	0.980493	0.581820	0.346427	0.785040	0.647973	.62806648	.66737844
PCT	9.40	0.364130	0.042330	0.010325	0.157724	0.981258	0.965021	0.990036	0.563962	0.244189	0.838128	0.641485	0.627613	0.655123
PCT 72h	9.50	0.364130	0.084160	0.031197	0.207754	0.965361	0.939219	0.980493	0.581820	0.346427	0.785040	0.647973	.62806648	.66737844
PCT	9.50	0.364130	0.042330	0.010325	0.157724	0.981258	0.965021	0.990036	0.563962	0.244189	0.838128	0.641485	0.627613	0.655123
PCT 72h	9.60	0.364130	0.084160	0.031197	0.207754	0.965361	0.939219	0.980493	0.581820	0.346427	0.785040	0.647973	.62806648	.66737844
PCT	9.60	0.364130	0.042330	0.010325	0.157724	0.981258	0.965021	0.990036	0.563962	0.244189	0.838128	0.641485	0.627613	0.655123
PCT 72h	9.70	0.364130	0.084160	0.031197	0.207754	0.965361	0.939219	0.980493	0.581820	0.346427	0.785040	0.647973	.62806648	.66737844
PCT	9.70	0.364130	0.042330	0.010325	0.157724	0.981258	0.965021	0.990036	0.563962	0.244189	0.838128	0.641485	0.627613	0.655123
PCT 72h	9.80	0.364130	0.084160	0.031197	0.207754	0.965361	0.939219	0.980493	0.581820	0.346427	0.785040	0.647973	.62806648	.66737844
PCT	9.80	0.364130	0.042330	0.010325	0.157724	0.981258	0.965021	0.990036	0.563962	0.244189	0.838128	0.641485	0.627613	0.655123
PCT 72h	9.90	0.364130	0.084160	0.031197	0.207754	0.965361	0.939219	0.980493	0.581820	0.346427	0.785040	0.647973	.62806648	.66737844
PCT	9.90	0.364130	0.042330	0.010325	0.157724	0.981258	0.965021	0.990036	0.563962	0.244189	0.838128	0.641485	0.627613	0.655123
PCT 72h	10.00	0.364130	0.062636	0.019844	0.180694	0.968024	0.943190	0.982207	0.528689	0.267161	0.775362	0.643289	.62622361	.65999762
PCT	10.00	0.364130	0.042330	0.010325	0.157724	0.982571	0.966725	0.990942	0.581732	0.257242	0.848145	0.641792	0.627931	0.655420
PCT 72h	10.10	0.364130	0.062636	0.019844	0.180694	0.971555	0.947969	0.984623	0.557716	0.289915	0.795693	0.644124	.62708837	.66080181
PCT	10.10	0.364130	0.042330	0.010325	0.157724	0.984343	0.968835	0.992196	0.607558	0.277234	0.862041	0.642206	0.628360	0.655819
PCT 72h	10.20	0.364130	0.062636	0.019844	0.180694	0.974648	0.952039	0.986747	0.585887	0.313284	0.814390	0.644852	.62784245	.66150298
PCT	10.20	0.364130	0.042330	0.010325	0.157724	0.985749	0.970337	0.993210	0.629763	0.295469	0.873401	0.642534	0.628699	0.656135
PCT 72h	10.30	0.364130	0.062636	0.019844	0.180694	0.974648	0.952039	0.986747	0.585887	0.313284	0.814390	0.644852	.62784245	.66150298
PCT	10.30	0.364130	0.042330	0.010325	0.157724	0.985749	0.970337	0.993210	0.629763	0.295469	0.873401	0.642534	0.628699	0.656135
PCT 72h	10.40	0.364130	0.062636	0.019844	0.180694	0.974858	0.952266	0.986904	0.587905	0.315009	0.815692	0.644901	.62789357	.66155051
PCT	10.40	0.364130	0.042330	0.010325	0.157724	0.985998	0.970590	0.993389	0.633859	0.298946	0.875440	0.642592	0.628759	0.656191
PCT 72h	10.50	0.364130	0.062636	0.019844	0.180694	0.974858	0.952266	0.986904	0.587905	0.315009	0.815692	0.644901	.62789357	.66155051
PCT	10.50	0.364130	0.042330	0.010325	0.157724	0.985998	0.970590	0.993389	0.633859	0.298946	0.875440	0.642592	0.628759	0.656191
PCT 72h	10.60	0.364130	0.062636	0.019844	0.180694	0.974858	0.952266	0.986904	0.587905	0.315009	0.815692	0.644901	.62789357	.66155051
PCT	10.60	0.364130	0.042330	0.010325	0.157724	0.985998	0.970590	0.993389	0.633859	0.298946	0.875440	0.642592	0.628759	0.656191
PCT 72h	10.70	0.364130	0.042330	0.010325	0.157724	0.974858	0.952266	0.986904	0.490867	0.195761	0.792477	0.639978	.62605387	.65367082
PCT	10.70	0.364130	0.042330	0.010325	0.157724	0.985998	0.970590	0.993389	0.633859	0.298946	0.875440	0.642592	0.628759	0.656191
PCT 72h	10.80	0.364130	0.042330	0.010325	0.157724	0.974858	0.952266	0.986904	0.490867	0.195761	0.792477	0.639978	.62605387	.65367082
PCT	10.80	0.364130	0.042330	0.010325	0.157724	0.985998	0.970590	0.993389	0.633859	0.298946	0.875440	0.642592	0.628759	0.656191
PCT 72h	10.90	0.364130	0.042330	0.010325	0.157724	0.974858	0.952266	0.986904	0.490867	0.195761	0.792477	0.639978	.62605387	.65367082
PCT	10.90	0.364130	0.042330	0.010325	0.157724	0.985998	0.970590	0.993389	0.633859	0.298946	0.875440	0.642592	0.628759	0.656191
PCT 72h	11.00	0.364130	0.042330	0.010325	0.157724	0.974858	0.952266	0.986904	0.490867	0.195761	0.792477	0.639978	.62605387	.65367082
PCT	11.00	0.364130	0.042330	0.010325	0.157724	0.985998	0.970590	0.993389	0.633859	0.298946	0.875440	0.642592	0.628759	0.656191
PCT 72h	11.10	0.364130	0.042330	0.010325	0.157724	0.974858	0.952266	0.986904	0.490867	0.195761	0.792477	0.639978	.62605387	.65367082
PCT	11.10	0.364130	0.042330	0.010325	0.157724	0.985998	0.970590	0.993389	0.633859	0.298946	0.875440	0.642592	0.628759	0.656191
PCT 72h	11.20	0.364130	0.042330	0.010325	0.157724	0.974858	0.952266	0.986904	0.490867	0.195761	0.792477	0.639978	.62605387	.65367082
PCT	11.20	0.364130	0.042330	0.010325	0.157724	0.985998	0.970590	0.993389	0.633859	0.298946	0.875440	0.642592	0.628759	0.656191
PCT 72h	11.30	0.364130	0.042330	0.010325	0.157724	0.975326	0.952526	0.987322	0.495569	0.198646	0.795651	0.640089	.62616845	.65377759
PCT	11.30	0.364130	0.042330	0.010325	0.157724	0.986137	0.970542	0.993532	0.636179	0.300931	0.876588	0.642624	0.628793	0.656223
PCT 72h	11.40	0.364130	0.042330	0.010325	0.157724	0.975326	0.952526	0.987322	0.495569	0.198646	0.795651	0.640089	.62616845	.65377759
PCT	11.40	0.364130	0.042330	0.010325	0.157724	0.986137	0.970542	0.993532	0.636179	0.300931	0.876588	0.642624	0.628793	0.656223
PCT 72h	11.50	0.364130	0.042330	0.010325	0.157724	0.975326	0.952526	0.987322	0.495569	0.198646	0.795651	0.640089	.62616845	.65377759

PCT	11.50	0.364130	0.042330	0.010325	0.157724	0.986137	0.970542	0.993532	0.636179	0.300931	0.876588	0.642624	0.628793	0.656223
PCT 72h	11.60	0.364130	0.042330	0.010325	0.157724	0.975326	0.952526	0.987322	0.495569	0.198646	0.795651	0.640089	.62616845	.65377759
PCT	11.60	0.364130	0.042330	0.010325	0.157724	0.986137	0.970542	0.993532	0.636179	0.300931	0.876588	0.642624	0.628793	0.656223
PCT 72h	11.70	0.364130	0.042330	0.010325	0.157724	0.975326	0.952526	0.987322	0.495569	0.198646	0.795651	0.640089	.62616845	.65377759
PCT	11.70	0.364130	0.042330	0.010325	0.157724	0.986137	0.970542	0.993532	0.636179	0.300931	0.876588	0.642624	0.628793	0.656223
PCT 72h	11.80	0.364130	0.042330	0.010325	0.157724	0.975326	0.952526	0.987322	0.495569	0.198646	0.795651	0.640089	.62616845	.65377759
PCT	11.80	0.364130	0.042330	0.010325	0.157724	0.986137	0.970542	0.993532	0.636179	0.300931	0.876588	0.642624	0.628793	0.656223
PCT 72h	11.90	0.364130	0.042330	0.010325	0.157724	0.975326	0.952526	0.987322	0.495569	0.198646	0.795651	0.640089	.62616845	.65377759
PCT	11.90	0.364130	0.042330	0.010325	0.157724	0.986137	0.970542	0.993532	0.636179	0.300931	0.876588	0.642624	0.628793	0.656223
PCT 72h	12.00	0.364130	0.042330	0.010325	0.157724	0.975326	0.952526	0.987322	0.495569	0.198646	0.795651	0.640089	.62616845	.65377759
PCT	12.00	0.364130	0.042330	0.010325	0.157724	0.986137	0.970542	0.993532	0.636179	0.300931	0.876588	0.642624	0.628793	0.656223
PCT 72h	12.10	0.364130	0.042330	0.010325	0.157724	0.975326	0.952526	0.987322	0.495569	0.198646	0.795651	0.640089	.62616845	.65377759
PCT	12.10	0.364130	0.042330	0.010325	0.157724	0.986137	0.970542	0.993532	0.636179	0.300931	0.876588	0.642624	0.628793	0.656223
PCT 72h	12.20	0.364130	0.042330	0.010325	0.157724	0.975326	0.952526	0.987322	0.495569	0.198646	0.795651	0.640089	.62616845	.65377759
PCT	12.20	0.364130	0.042330	0.010325	0.157724	0.986137	0.970542	0.993532	0.636179	0.300931	0.876588	0.642624	0.628793	0.656223
PCT 72h	12.30	0.364130	0.042330	0.010325	0.157724	0.975326	0.952526	0.987322	0.495569	0.198646	0.795651	0.640089	.62616845	.65377759
PCT	12.30	0.364130	0.042330	0.010325	0.157724	0.986137	0.970542	0.993532	0.636179	0.300931	0.876588	0.642624	0.628793	0.656223
PCT 72h	12.40	0.364130	0.042330	0.010325	0.157724	0.975326	0.952526	0.987322	0.495569	0.198646	0.795651	0.640089	.62616845	.65377759
PCT	12.40	0.364130	0.042330	0.010325	0.157724	0.986137	0.970542	0.993532	0.636179	0.300931	0.876588	0.642624	0.628793	0.656223
PCT 72h	12.50	0.364130	0.042330	0.010325	0.157724	0.977338	0.954613	0.988818	0.516821	0.212057	0.809566	0.640563	.62665949	.65423514
PCT	12.50	0.364130	0.042330	0.010325	0.157724	0.987144	0.971339	0.994285	0.653441	0.316080	0.884958	0.642859	0.629035	0.656449
PCT 72h	12.60	0.364130	0.042330	0.010325	0.157724	0.977338	0.954613	0.988818	0.516821	0.212057	0.809566	0.640563	.62665949	.65423514
PCT	12.60	0.364130	0.042330	0.010325	0.157724	0.987144	0.971339	0.994285	0.653441	0.316080	0.884958	0.642859	0.629035	0.656449
PCT 72h	12.70	0.364130	0.042330	0.010325	0.157724	0.977338	0.954613	0.988818	0.516821	0.212057	0.809566	0.640563	.62665949	.65423514
PCT	12.70	0.364130	0.042330	0.010325	0.157724	0.987144	0.971339	0.994285	0.653441	0.316080	0.884958	0.642859	0.629035	0.656449
PCT 72h	12.80	0.364130	0.042330	0.010325	0.157724	0.977338	0.954613	0.988818	0.516821	0.212057	0.809566	0.640563	.62665949	.65423514
PCT	12.80	0.364130	0.042330	0.010325	0.157724	0.987144	0.971339	0.994285	0.653441	0.316080	0.884958	0.642859	0.629035	0.656449
PCT 72h	12.90	0.364130	0.042330	0.010325	0.157724	0.977338	0.954613	0.988818	0.516821	0.212057	0.809566	0.640563	.62665949	.65423514
PCT	12.90	0.364130	0.042330	0.010325	0.157724	0.987144	0.971339	0.994285	0.653441	0.316080	0.884958	0.642859	0.629035	0.656449
PCT 72h	13.00	0.364130	0.042330	0.010325	0.157724	0.977867	0.954940	0.989260	0.522721	0.215893	0.813309	0.640688	.6267885	.65435535
PCT	13.00	0.364130	0.042330	0.010325	0.157724	0.987144	0.971339	0.994285	0.653441	0.316080	0.884958	0.642859	0.629035	0.656449
PCT 72h	13.10	0.364130	0.042330	0.010325	0.157724	0.978182	0.955721	0.989376	0.526293	0.218240	0.815551	0.640762	.62686518	.65442679
PCT	13.10	0.364130	0.042330	0.010325	0.157724	0.987849	0.973368	0.994501	0.666099	0.327630	0.890914	0.643023	0.629205	0.656607
PCT 72h	13.20	0.364130	0.042330	0.010325	0.157724	0.978182	0.955721	0.989376	0.526293	0.218240	0.815551	0.640762	.62686518	.65442679
PCT	13.20	0.364130	0.042330	0.010325	0.157724	0.987849	0.973368	0.994501	0.666099	0.327630	0.890914	0.643023	0.629205	0.656607
PCT 72h	13.30	0.364130	0.042330	0.010325	0.157724	0.978182	0.955721	0.989376	0.526293	0.218240	0.815551	0.640762	.62686518	.65442679
PCT	13.30	0.364130	0.042330	0.010325	0.157724	0.987849	0.973368	0.994501	0.666099	0.327630	0.890914	0.643023	0.629205	0.656607
PCT 72h	13.40	0.364130	0.042330	0.010325	0.157724	0.978182	0.955721	0.989376	0.526293	0.218240	0.815551	0.640762	.62686518	.65442679
PCT	13.40	0.364130	0.042330	0.010325	0.157724	0.987849	0.973368	0.994501	0.666099	0.327630	0.890914	0.643023	0.629205	0.656607
PCT 72h	13.50	0.364130	0.042330	0.010325	0.157724	0.978182	0.955721	0.989376	0.526293	0.218240	0.815551	0.640762	.62686518	.65442679
PCT	13.50	0.364130	0.042330	0.010325	0.157724	0.987849	0.973368	0.994501	0.666099	0.327630	0.890914	0.643023	0.629205	0.656607
PCT 72h	13.60	0.364130	0.042330	0.010325	0.157724	0.978182	0.955721	0.989376	0.526293	0.218240	0.815551	0.640762	.62686518	.65442679
PCT	13.60	0.364130	0.042330	0.010325	0.157724	0.987849	0.973368	0.994501	0.666099	0.327630	0.890914	0.643023	0.629205	0.656607
PCT 72h	13.70	0.364130	0.042330	0.010325	0.157724	0.978517	0.956336	0.989553	0.530145	0.220793	0.817948	0.640841	.62694666	.65450271
PCT	13.70	0.364130	0.042330	0.010325	0.157724	0.988306	0.974377	0.994704	0.674571	0.335581	0.894817	0.643129	0.629315	0.656709
PCT 72h	13.80	0.364130	0.042330	0.010325	0.157724	0.978517	0.956336	0.989553	0.530145	0.220793	0.817948	0.640841	.62694666	.65450271
PCT	13.80	0.364130	0.042330	0.010325	0.157724	0.988306	0.974377	0.994704	0.674571	0.335581	0.894817	0.643129	0.629315	0.656709

PCT 72h	13.90	0.364130	0.042330	0.010325	0.157724	0.978725	0.956527	0.989710	0.532570	0.222411	0.819446	0.640890	.62699733	.65454992
PCT	13.90	0.364130	0.042330	0.010325	0.157724	0.988542	0.974514	0.994890	0.679035	0.339843	0.896847	0.643184	0.629371	0.656762
PCT 72h	14.00	0.364130	0.042330	0.010325	0.157724	0.978725	0.956527	0.989710	0.532570	0.222411	0.819446	0.640890	.62699733	.65454992
PCT	14.00	0.364130	0.042330	0.010325	0.157724	0.988542	0.974514	0.994890	0.679035	0.339843	0.896847	0.643184	0.629371	0.656762
PCT 72h	14.10	0.364130	0.042330	0.010325	0.157724	0.978725	0.956527	0.989710	0.532570	0.222411	0.819446	0.640890	.62699733	.65454992
PCT	14.10	0.364130	0.042330	0.010325	0.157724	0.988542	0.974514	0.994890	0.679035	0.339843	0.896847	0.643184	0.629371	0.656762
PCT 72h	14.20	0.364130	0.042330	0.010325	0.157724	0.978725	0.956527	0.989710	0.532570	0.222411	0.819446	0.640890	.62699733	.65454992
PCT	14.20	0.364130	0.042330	0.010325	0.157724	0.988542	0.974514	0.994890	0.679035	0.339843	0.896847	0.643184	0.629371	0.656762
PCT 72h	14.30	0.364130	0.042330	0.010325	0.157724	0.978725	0.956527	0.989710	0.532570	0.222411	0.819446	0.640890	.62699733	.65454992
PCT	14.30	0.364130	0.042330	0.010325	0.157724	0.988542	0.974514	0.994890	0.679035	0.339843	0.896847	0.643184	0.629371	0.656762
PCT 72h	14.40	0.364130	0.042330	0.010325	0.157724	0.978725	0.956527	0.989710	0.532570	0.222411	0.819446	0.640890	.62699733	.65454992
PCT	14.40	0.364130	0.042330	0.010325	0.157724	0.988542	0.974514	0.994890	0.679035	0.339843	0.896847	0.643184	0.629371	0.656762
PCT 72h	14.50	0.364130	0.042330	0.010325	0.157724	0.978725	0.956527	0.989710	0.532570	0.222411	0.819446	0.640890	.62699733	.65454992
PCT	14.50	0.364130	0.042330	0.010325	0.157724	0.988542	0.974514	0.994890	0.679035	0.339843	0.896847	0.643184	0.629371	0.656762
PCT 72h	14.60	0.364130	0.042330	0.010325	0.157724	0.978725	0.956527	0.989710	0.532570	0.222411	0.819446	0.640890	.62699733	.65454992
PCT	14.60	0.364130	0.042330	0.010325	0.157724	0.988542	0.974514	0.994890	0.679035	0.339843	0.896847	0.643184	0.629371	0.656762
PCT 72h	14.70	0.364130	0.042330	0.010325	0.157724	0.978725	0.956527	0.989710	0.532570	0.222411	0.819446	0.640890	.62699733	.65454992
PCT	14.70	0.364130	0.042330	0.010325	0.157724	0.988542	0.974514	0.994890	0.679035	0.339843	0.896847	0.643184	0.629371	0.656762
PCT 72h	14.80	0.364130	0.042330	0.010325	0.157724	0.978725	0.956527	0.989710	0.532570	0.222411	0.819446	0.640890	.62699733	.65454992
PCT	14.80	0.364130	0.042330	0.010325	0.157724	0.988542	0.974514	0.994890	0.679035	0.339843	0.896847	0.643184	0.629371	0.656762
PCT 72h	14.90	0.364130	0.042330	0.010325	0.157724	0.978725	0.956527	0.989710	0.532570	0.222411	0.819446	0.640890	.62699733	.65454992
PCT	14.90	0.364130	0.042330	0.010325	0.157724	0.988542	0.974514	0.994890	0.679035	0.339843	0.896847	0.643184	0.629371	0.656762
PCT 72h	15.00	0.364130	0.042330	0.010325	0.157724	0.978725	0.956527	0.989710	0.532570	0.222411	0.819446	0.640890	.62699733	.65454992
PCT	15.00	0.364130	0.042330	0.010325	0.157724	0.988542	0.974514	0.994890	0.679035	0.339843	0.896847	0.643184	0.629371	0.656762
PCT 72h	15.10	0.364130	0.042330	0.010325	0.157724	0.978725	0.956527	0.989710	0.532570	0.222411	0.819446	0.640890	.62699733	.65454992
PCT	15.10	0.364130	0.042330	0.010325	0.157724	0.988542	0.974514	0.994890	0.679035	0.339843	0.896847	0.643184	0.629371	0.656762
PCT 72h	15.20	0.364130	0.042330	0.010325	0.157724	0.978725	0.956527	0.989710	0.532570	0.222411	0.819446	0.640890	.62699733	.65454992
PCT	15.20	0.364130	0.042330	0.010325	0.157724	0.988542	0.974514	0.994890	0.679035	0.339843	0.896847	0.643184	0.629371	0.656762
PCT 72h	15.30	0.364130	0.042330	0.010325	0.157724	0.978725	0.956527	0.989710	0.532570	0.222411	0.819446	0.640890	.62699733	.65454992
PCT	15.30	0.364130	0.042330	0.010325	0.157724	0.988542	0.974514	0.994890	0.679035	0.339843	0.896847	0.643184	0.629371	0.656762
PCT 72h	15.40	0.364130	0.042330	0.010325	0.157724	0.978725	0.956527	0.989710	0.532570	0.222411	0.819446	0.640890	.62699733	.65454992
PCT	15.40	0.364130	0.042330	0.010325	0.157724	0.988542	0.974514	0.994890	0.679035	0.339843	0.896847	0.643184	0.629371	0.656762
PCT 72h	15.50	0.364130	0.042330	0.010325	0.157724	0.978725	0.956527	0.989710	0.532570	0.222411	0.819446	0.640890	.62699733	.65454992
PCT	15.50	0.364130	0.042330	0.010325	0.157724	0.988542	0.974514	0.994890	0.679035	0.339843	0.896847	0.643184	0.629371	0.656762
PCT 72h	15.60	0.364130	0.020998	0.002857	0.138328	0.980035	0.957605	0.990713	0.375880	0.078269	0.810300	0.636114	.62634649	.64576964
PCT	15.60	0.364130	0.020998	0.002857	0.138328	0.988542	0.974514	0.994890	0.512063	0.126616	0.883679	0.638112	0.628420	0.647693
PCT 72h	15.70	0.364130	0.020998	0.002857	0.138328	0.980035	0.957605	0.990713	0.375880	0.078269	0.810300	0.636114	.62634649	.64576964
PCT	15.70	0.364130	0.020998	0.002857	0.138328	0.988542	0.974514	0.994890	0.512063	0.126616	0.883679	0.638112	0.628420	0.647693
PCT 72h	15.80	0.364130	0.020998	0.002857	0.138328	0.980035	0.957605	0.990713	0.375880	0.078269	0.810300	0.636114	.62634649	.64576964
PCT	15.80	0.364130	0.020998	0.002857	0.138328	0.988542	0.974514	0.994890	0.512063	0.126616	0.883679	0.638112	0.628420	0.647693
PCT 72h	15.90	0.364130	0.020998	0.002857	0.138328	0.980035	0.957605	0.990713	0.375880	0.078269	0.810300	0.636114	.62634649	.64576964
PCT	15.90	0.364130	0.020998	0.002857	0.138328	0.988542	0.974514	0.994890	0.512063	0.126616	0.883679	0.638112	0.628420	0.647693
PCT 72h	16.00	0.364130	0.020998	0.002857	0.138328	0.980035	0.957605	0.990713	0.375880	0.078269	0.810300	0.636114	.62634649	.64576964
PCT	16.00	0.364130	0.020998	0.002857	0.138328	0.988542	0.974514	0.994890	0.512063	0.126616	0.883679	0.638112	0.628420	0.647693
PCT 72h	16.10	0.364130	0.020998	0.002857	0.138328	0.980035	0.957605	0.990713	0.375880	0.078269	0.810300	0.636114	.62634649	.64576964
PCT	16.10	0.364130	0.020998	0.002857	0.138328	0.988542	0.974514	0.994890	0.512063	0.126616	0.883679	0.638112	0.628420	0.647693
PCT 72h	16.20	0.364130	0.020998	0.002857	0.138328	0.980035	0.957605	0.990713	0.375880	0.078269	0.810300	0.636114	.62634649	.64576964

PCT	16.20	0.364130	0.020998	0.002857	0.138328	0.988542	0.974514	0.994890	0.512063	0.126616	0.883679	0.638112	0.628420	0.647693
PCT 72h	16.30	0.364130	0.020998	0.002857	0.138328	0.980035	0.957605	0.990713	0.375880	0.078269	0.810300	0.636114	.62634649	.64576964
PCT	16.30	0.364130	0.020998	0.002857	0.138328	0.988542	0.974514	0.994890	0.512063	0.126616	0.883679	0.638112	0.628420	0.647693
PCT 72h	16.40	0.364130	0.020998	0.002857	0.138328	0.980035	0.957605	0.990713	0.375880	0.078269	0.810300	0.636114	.62634649	.64576964
PCT	16.40	0.364130	0.020998	0.002857	0.138328	0.988542	0.974514	0.994890	0.512063	0.126616	0.883679	0.638112	0.628420	0.647693
PCT 72h	16.50	0.364130	0.020998	0.002857	0.138328	0.980035	0.957605	0.990713	0.375880	0.078269	0.810300	0.636114	.62634649	.64576964
PCT	16.50	0.364130	0.020998	0.002857	0.138328	0.988542	0.974514	0.994890	0.512063	0.126616	0.883679	0.638112	0.628420	0.647693
PCT 72h	16.60	0.364130	0.020998	0.002857	0.138328	0.980035	0.957605	0.990713	0.375880	0.078269	0.810300	0.636114	.62634649	.64576964
PCT	16.60	0.364130	0.020998	0.002857	0.138328	0.988542	0.974514	0.994890	0.512063	0.126616	0.883679	0.638112	0.628420	0.647693
PCT 72h	16.70	0.364130	0.020998	0.002857	0.138328	0.980035	0.957605	0.990713	0.375880	0.078269	0.810300	0.636114	.62634649	.64576964
PCT	16.70	0.364130	0.020998	0.002857	0.138328	0.988542	0.974514	0.994890	0.512063	0.126616	0.883679	0.638112	0.628420	0.647693
PCT 72h	16.80	0.364130	0.020998	0.002857	0.138328	0.980035	0.957605	0.990713	0.375880	0.078269	0.810300	0.636114	.62634649	.64576964
PCT	16.80	0.364130	0.020998	0.002857	0.138328	0.988542	0.974514	0.994890	0.512063	0.126616	0.883679	0.638112	0.628420	0.647693
PCT 72h	16.90	0.364130	0.020998	0.002857	0.138328	0.980035	0.957605	0.990713	0.375880	0.078269	0.810300	0.636114	.62634649	.64576964
PCT	16.90	0.364130	0.020998	0.002857	0.138328	0.988542	0.974514	0.994890	0.512063	0.126616	0.883679	0.638112	0.628420	0.647693
PCT 72h	17.00	0.364130	0.020998	0.002857	0.138328	0.980035	0.957605	0.990713	0.375880	0.078269	0.810300	0.636114	.62634649	.64576964
PCT	17.00	0.364130	0.020998	0.002857	0.138328	0.988542	0.974514	0.994890	0.512063	0.126616	0.883679	0.638112	0.628420	0.647693
